# Myocardial bridging is an independent predictor of positive spasm provocation testing by intracoronary ergonovine injections: a retrospective observational study

**DOI:** 10.1007/s00380-019-01518-7

**Published:** 2019-09-27

**Authors:** Riku Arai, Hiroto Kano, Shinya Suzuki, Hiroaki Semba, Takuto Arita, Naoharu Yagi, Takayuki Otsuka, Shunsuke Matsuno, Minoru Matsuhama, Yuko Kato, Tokuhisa Uejima, Yuji Oikawa, Yasuo Okumura, Junji Yajima, Takeshi Yamashita

**Affiliations:** 1 Department of Cardiovascular Medicine, The Cardiovascular Institute, Nishiazabu 3-2-19, Minato-ku, Tokyo, 1060031 Japan; 2 Division of Cardiology, Department of Medicine, Nihon University School of Medicine, Tokyo, Japan

**Keywords:** Myocardial bridging, Spasm provocation test, Ergonovine, Coronary spasm

## Abstract

The relationship between myocardial bridging (MB) and coronary spasms during spasm provocation testing (SPT) remains unclear. We aimed to investigate whether MB was correlated with the SPT by ergonovine (ER) injections in a retrospective observational study. Of the 3340 patients who underwent a first coronary angiography, 166 underwent SPT using ER injections and were divided into 2 groups: MB(+) (*n* = 23), and MB(−) (*n* = 143). MB was defined as an angiographic reduction in the diameter of the coronary artery during systole. The patients who had severe organic stenosis in the left anterior descending coronary artery were excluded. The MB(+) group more frequently had diabetes mellitus and chronic kidney disease, and a thicker interventricular septum thickness. The rate of SPT-positivity was higher in the MB(+) group than MB(−) group (56.5% vs. 22.4%, *P* = 0.001). A multivariate regression analysis showed that the presence of MB was independently associated with SPT-positivity (odds ratio 5.587, 95% confidence interval 2.061–15.149, *P* = 0.001). In conclusion, coronary spasms during provocation tests with ER independently correlated with the MB. MB may predict coronary spasms.

## Introduction

Myocardial bridging (MB) is an anomaly characterized by epicardial coronary vessels diving into the myocardium. MB is reflected as “squeezing” in coronary angiography (CAG). The frequency of MB has been reported in 0.5–12% of CAG images [1, 2]. Although MB has been considered as a benign condition, several complications have been reported, including ischemic chest pain [3], acute coronary syndrome (ACS) [4], sudden cardiac death [5], syncope [6], and coronary spasms [7]. Several reports have suggested that MB increases the risk of coronary spasms, especially in the MB segments because the longstanding compression–relaxation effects of MB may induce focal endothelial dysfunction by direct stress on the endothelium with enhanced vasoreactivity [7–10]. However, Sara et al. reported that MB was significantly associated with not only focal (epicardial) but also microvascular endothelial dysfunction in patients with non-obstructive coronary arteries [11]. Nevertheless, although there is a pathophysiological relationship between MB and endothelial dysfunction, little is known as to whether MB is associated with coronary spasms during spasm provocation testing (SPT) [12]. Therefore, we studied whether MB was related to coronary spasms by SPT using intracoronary ergonovine (ER) injections in a retrospective observational study.

## Methods

### Study participants and protocol

**Fig. 1:**
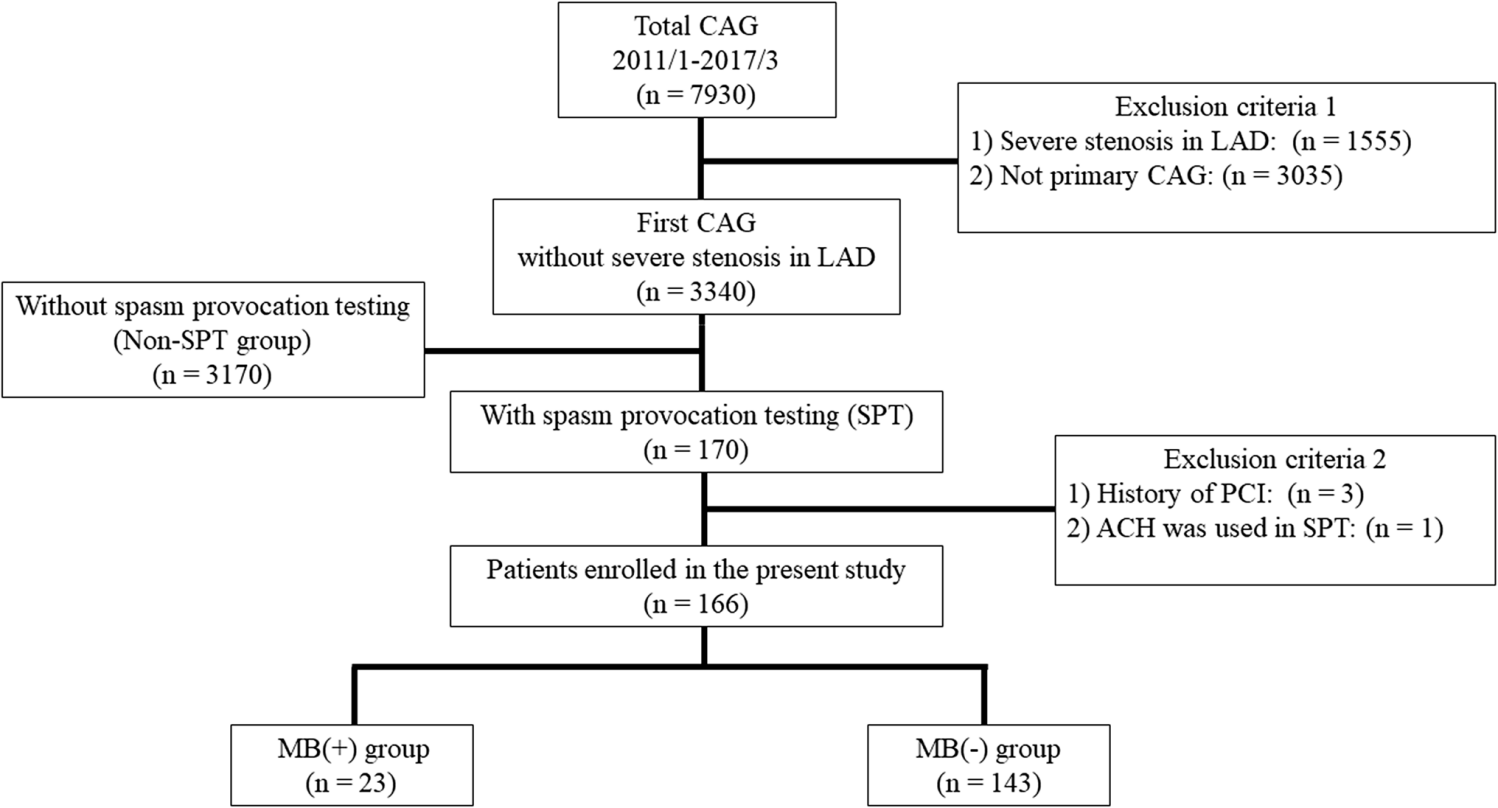
Flowchart for the patient selection. *CAG* coronary angiography, *SPT* spasm provocation test, *LAD* left anterior descending coronary artery, *PCI* percutaneous coronary intervention, *MB* myocardial bridging, *ACH* acetylcholine

Between January 2011 and March 2017, 7930 patients underwent CAG at the Cardiovascular Institute Hospital in Tokyo, Japan. The flowchart for the patient selection is shown in Fig. 1. The first set of exclusion criteria were as follows: (1) severe stenosis (as subtotal or total occlusion) in the left anterior descending coronary artery (LAD), in which MB was mostly involved [1] and (2) CAG more than once in this hospital, i.e., this was not the first CAG. Then, 3340 patients who underwent a first CAG without any severe organic stenosis in the LAD were enrolled. To investigate the relationship between MB and coronary spasms, we selected 170 patients who underwent SPT by intracoronary ER injections. We first evaluated how to select the patients who underwent SPT (SPT group). Among the 3340 patients who had a first CAG without any severe stenosis in the LAD, we compared the patient characteristics between the 170 SPT patients and remaining 3170 patients who did not undergo SPT (non-SPT group). The second set of exclusion criteria was as follows: (1) a history of percutaneous coronary intervention (PCI) and (2) acetylcholine (ACH) used in the SPT. Finally, a total of 166 patients were retrospectively analysed. The presence of MB was defined as an angiographic reduction in the diameter of the coronary artery during systole, also known as the “milking effect’’ between systole and diastole [13].

The study protocol complied with the Declaration of Helsinki. Written informed consent for the CAG and SPT was obtained from all patients and the ethics committee at the Cardiovascular Institute approved the study protocol.

### Data collection on admission for the first CAG

For each patient, the cardiovascular status was evaluated using echocardiography and serum tests on admission for the first CAG. The cardiovascular risk factors were defined as follows: hypertension (use of antihypertensive agents, systolic blood pressure ≥ 140 mmHg, or diastolic blood pressure ≥ 90 mmHg), diabetes mellitus (use of oral hypoglycaemic agents or insulin, or glycosylated hemoglobin ≥ 6.5%), dyslipidemia (use of statins or drugs for lowering triglyceride, low-density lipoprotein ≥ 140 mg/dl, high-density lipoprotein < 40 mg/dl, or triglyceride ≥ 150 mg/dl), and chronic kidney disease [estimated glomerular filtration rate (eGFR) < 60 ml/min/m^2^]. Information regarding the medications included the use of antihypertensive agents, beta blockers, calcium channel blockers, renin–angiotensin system inhibitors, antiplatelet medications, and anticoagulants. The eGFR was calculated using the equation for the Japanese population: eGFR = 194 × (serum creatinine)^–1.094^ × (age)^–0.287^ × (0.739 for female patients).

### Spasm provocation test (SPT) using intracoronary ergonovine injections

Experienced cardiologists performed the CAG via a transradial or transfemoral approach based on the current guidelines. Patients were instructed to stop using vasodilators, including calcium channel blockers and nitrates at least 24 h prior to the CAG and SPT. Diagnostic coronary angiograms were obtained of the right coronary artery (RCA) first, and then, of the left coronary artery (LCA). If coronary lesions with organic stenosis [defined as > 75% luminal narrowing according to the American College of Cardiology (ACC)/American Heart Association (AHA) classification [14]] were not observed, SPT was performed of the LCA and RCA using intracoronary ER injections. ER (20 μg, 0.2 mg/ml) in a 0.9% saline solution was injected up to 3 times per LCA and RCA. The SPT was first performed from the LCA because the catheter was still placed in the LCA after the diagnostic coronary angiogram. Standard 12-lead electrocardiograms (ECGs) were recorded carefully. At 1.5 min after each intracoronary injection of ER or when usual chest pain or significant ischemic ST changes on the electrocardiogram appeared, we obtained coronary angiograms. When the SPT of LCA was negative or symptoms and ECG changes improved rapidly even if the SPT of LCA was positive, SPT of RCA was continued. If symptoms and ECG changes persisted after the SPT of LCA, we injected nitrates intracoronary at that point and did not perform the SPT of RCA. After the SPT was completed, intracoronary nitrates were injected to reverse the coronary vasospasms. SPT-positivity was defined as a > 90% transient luminal narrowing, and typical chest symptoms, or significant ischemic ECG changes, according to the Japanese Circulation Society guidelines [15]. A positive criterion of 90% > stenosis was defined as severe stenosis with an angiographically visual contrast delay rather than quantitative coronary angiography (QCA) in the present study. SPT-negativity was defined as a case in which the positive criteria were not met even after 3 ER injections in both the LCA and RCA. The significant ischemic ECG changes were as follows: (1) ST-segment elevation of > 0.1 mV in at least two contiguous leads, (2) ST-segment depression of ≥ 0.1 mV in at least two contiguous leads, or (3) negative *U* waves.

### Representative patients

**Fig. 2:**
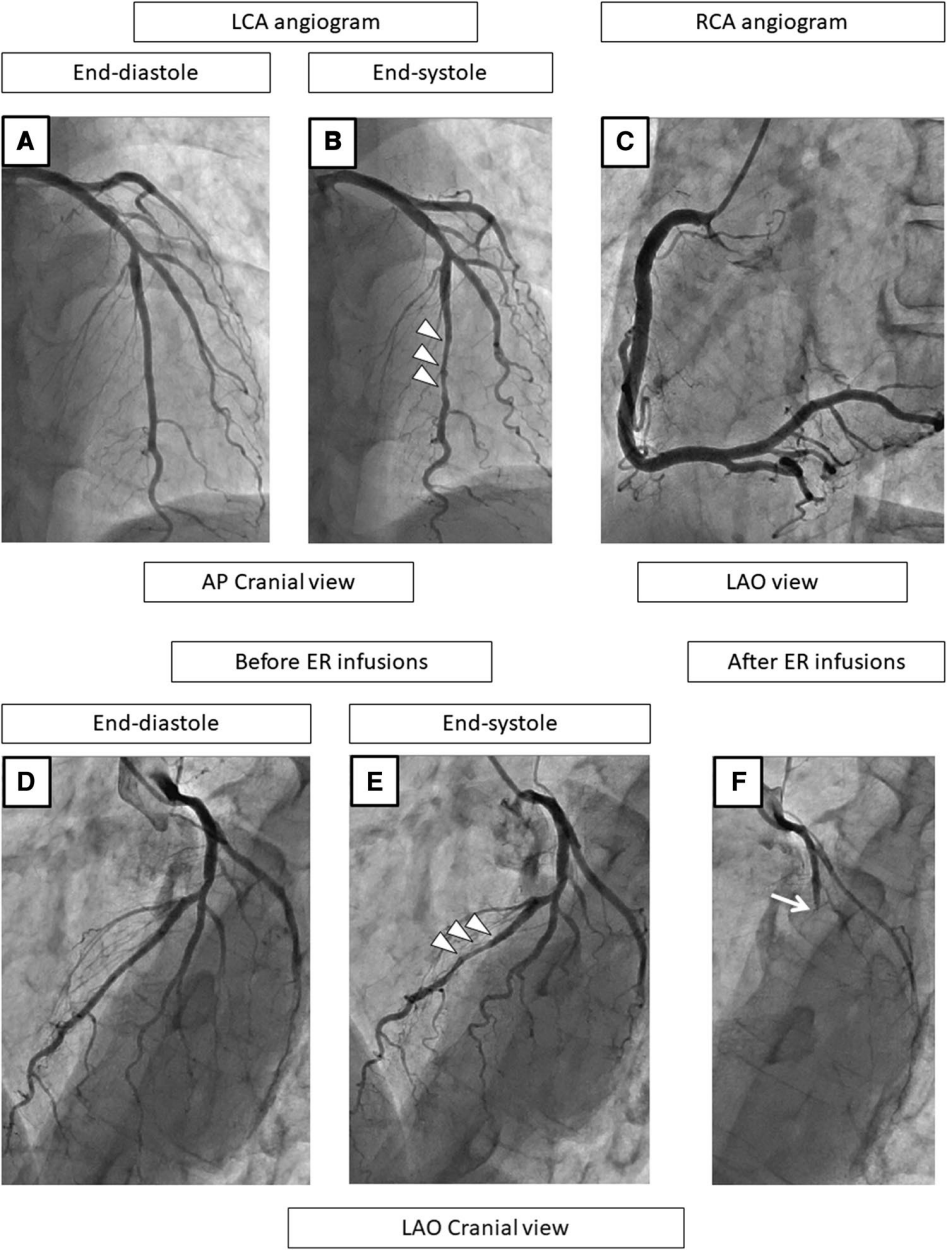
A representative case with MB and SPT-positivity in the LAD on the coronary angiogram at baseline (**a**–**c**), before (**d**, **e**), and after the SPT (**f**). At baseline and before the ER injections, the LCA angiogram shows no stenosis during the end-diastole phase (**a**, **d**) but MB is observed in the mid-LAD, as shown by squeezing (arrow heads) during the end-systole phase (**b**, **e**). The RCA angiogram shows no organic stenosis (**c**). After the ER injections, the LAD becomes a total occlusion at the proximal lesion (**f**). After the ER injections into the LCA, the chest pain and ECG changes persist and nitrates are required to relieve the vasospasms, and therefore, SPT in the RCA was not performed. In this case, the culprit vessel of the SPT-positivity was the LAD, and the positional relationship of the vasospasms to the MB was the “segment proximal to the MB” (**f**). *CAG* coronary angiography, *SPT* spasm provocation test, *ER* ergonovine, *LAD* left anterior descending coronary artery, *RCA* right coronary artery, *ECG* electrocardiogram

**Fig. 3:**
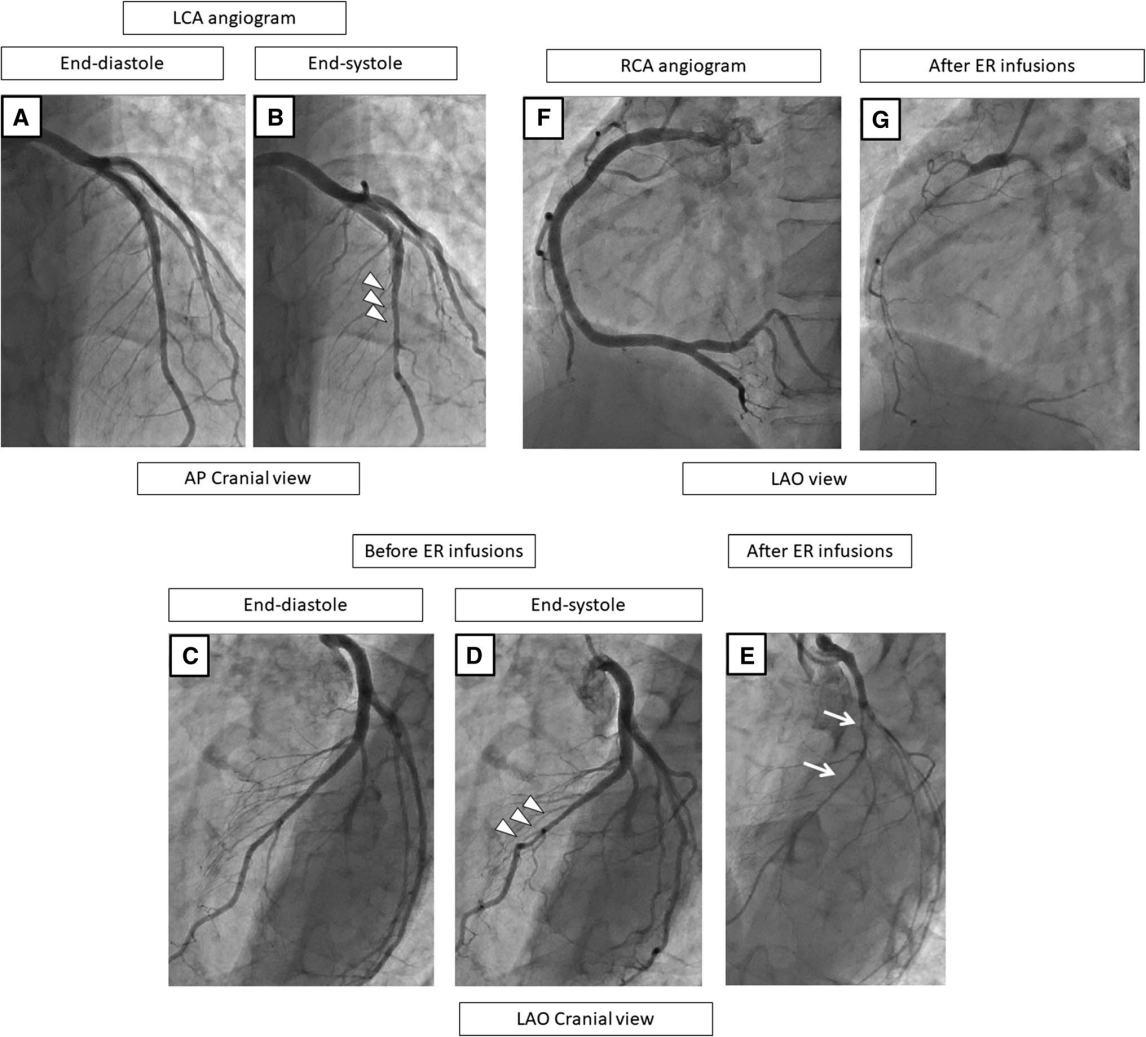
A representative case with MB and a spastic vessel (SPT-negativity) in the LAD but with SPT-positivity in the RCA on the coronary angiogram at baseline (**a**, **b**), before (**c**, **d**, **f**) and after the SPT (**e**, **g**). At baseline and before the ER injections, the LCA angiogram shows no organic stenosis during the end-diastole phase (**a**, **c**), but MB in the mid-LAD is observed as shown by the squeezing (arrow heads) during the end-systole phase (**b**, **d**). After the ER injections in the LCA, the LAD exhibits severe stenosis without a delay (**e**), but no ECG changes or chest symptoms occur. Before the ER injections into the RCA, no stenosis is observed (**f**), but the ER injections cause a subtotal occlusion (**g**). In this case, the culprit vessel of the SPT-positivity was the RCA, even though the LAD was a spastic vessel, suggesting a certain degree of endothelial dysfunction in the LAD. *CAG* coronary angiography, *SPT* spasm provocation test, *ER* ergonovine, *LAD* left anterior descending coronary artery, *LCA* left coronary artery, *RCA* right coronary artery, *ECG* electrocardiogram

**Fig. 4:**
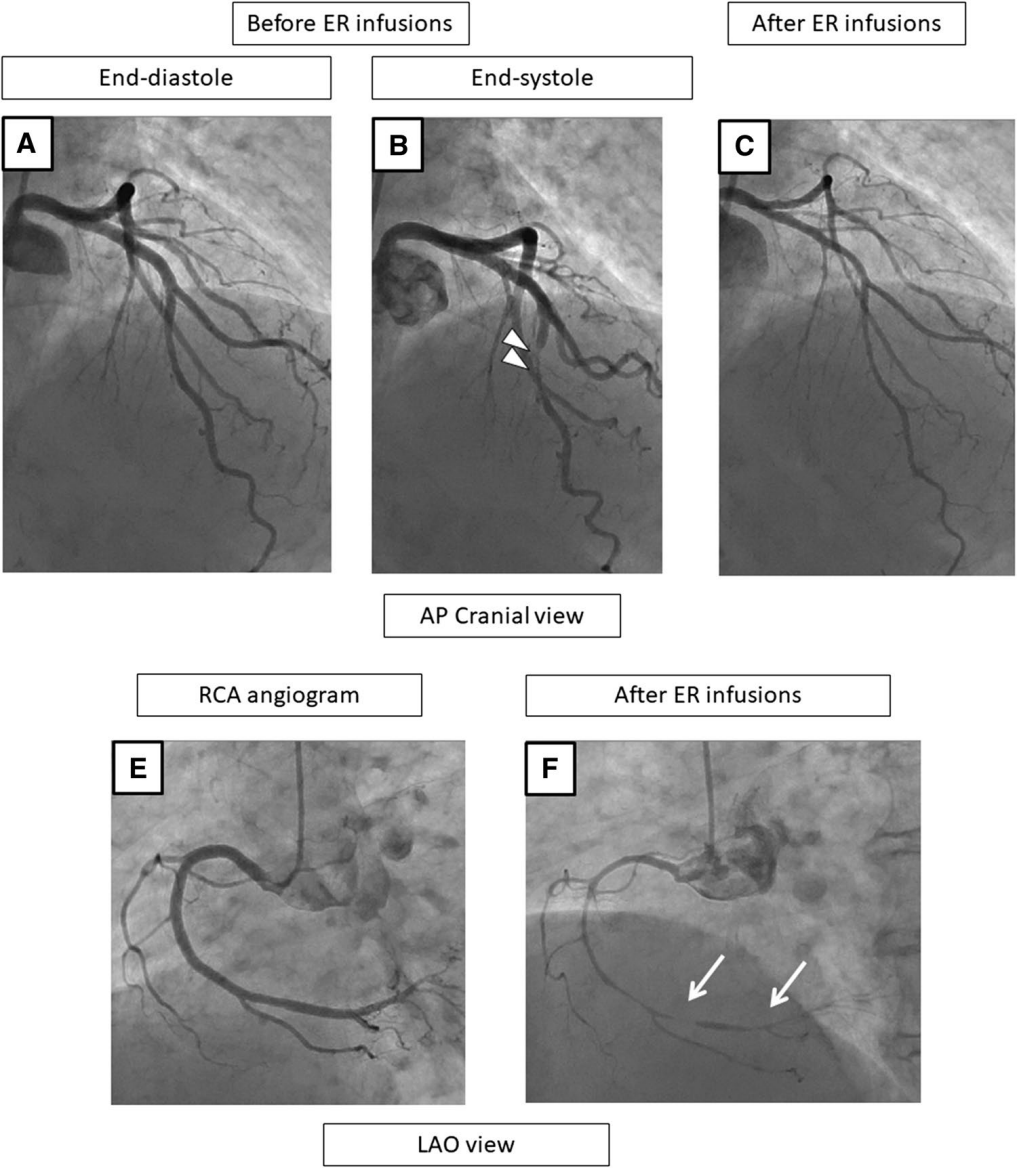
A representative case with MB and SPT-negativity in the LAD but with SPT-positivity in the RCA on the coronary angiogram before (**a**–**c**) and after the SPT (**c**, **f**). Before the ER injections, the LCA angiogram shows no significant stenosis during end-diastole (**a**) but MB in the mid-LAD is observed as shown by the “squeezing” (arrow heads) (**b**). After the ER injections into the LCA, the LAD does not have any spastic changes (**c**). Before the ER injection in the RCA, no stenosis was observed (**e**), but the ER injections cause a subtotal occlusion (**f**). In this case, the culprit vessel of the SPT-positivity was the RCA. *CAG* coronary angiography, *SPT* spasm provocation test, *ER* ergonovine, *LCA* left coronary artery, *LAD* left anterior descending coronary artery, *RCA* right coronary artery

**Fig. 5:**
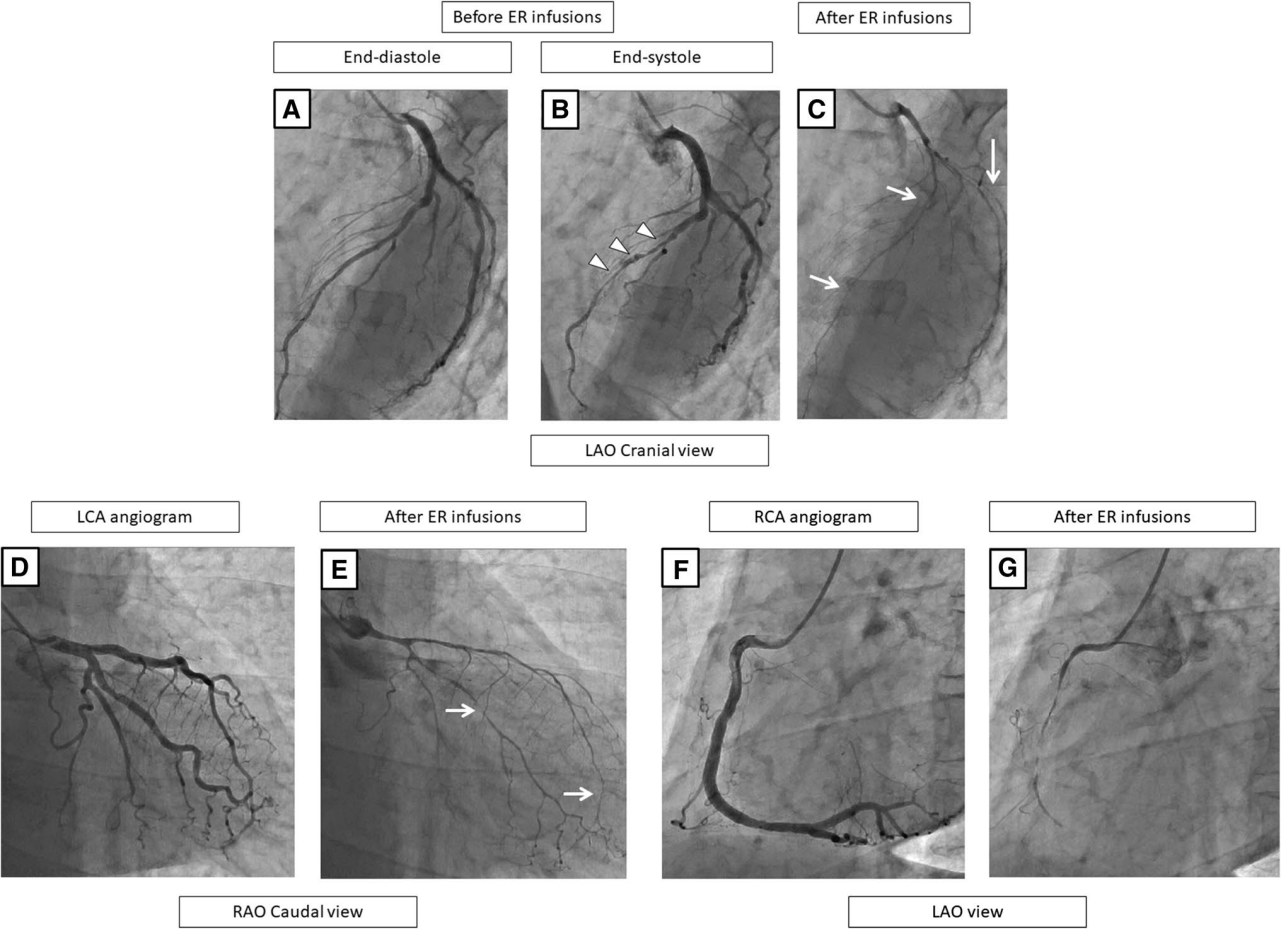
A representative case with diffuse MB in the LAD and multivessel spasms on the coronary angiogram before (**a**, **b**, **f**) and after the SPT (**c**–**e**, **g**). Before the ER injections, the LCA exhibits no stenosis during end-diastole (**a**), but MB in the mid-LAD is observed as shown by the “squeezing” (arrow head) (**b**). After the ER injections in the LCA, both the LAD and LCX show a subtotal occlusion with a delay (**c**, **e**), and chest pain and ECG changes occur. Thereafter, the chest pain and ECG changes improved without using nitrate infusions (**d**), so the SPT of the RCA was continued and no significant stenosis was observed (**f**). The ER injections in the RCA cause a total occlusion (**g**). In this case, the culprit vessels of the SPT-positivity were the RCA, LAD, and LCX. The positional relationship of the vasospasms to the MB segment was “diffuse, namely the segments proximal to the MB, on the MB, and distal to the MB” (**c**, **e**). *CAG* coronary angiography, *SPT* spasm provocation test, *ER* ergonovine, *LCA* left coronary artery, *LAD* left anterior descending coronary artery, *RCA* right coronary artery, *LCX* left circumflex artery, *ECG* electrocardiogram

Representative patients are shown in Figs. 2, 3, 4, and 5.

### Statistical analysis

All analyses were performed using SPSS version 19.0 software (SPSS Inc., Chicago, IL, USA). In all analyses, a *P* < 0.05 was taken to indicate statistical significance.

The patients were divided into two groups according to the presence of MB [MB(+) group; *n* = 23, and MB(−) group; *n* = 143). The patient background was compared between the two groups. The differences in the categorical and consecutive variables were tested using the Chi-square and Student’s *t* tests, respectively. Then, univariate logistic regression analyses were conducted to identify the candidate variables among the clinical, laboratory, and echocardiography parameters. The multivariate model included the variables with a significance level of *P *< 0.005 in the univariate analysis. We then performed a multivariate logistic regression analysis using stepwise methods.

## Results

### Patient characteristics between the SPT group and non-SPT group

The patient characteristics between the SPT group and non-SPT group are shown in Table 1. In the SPT group, the age was younger and comprised of males, cardiovascular risks, comorbidities, and organic heart disease were less prevalent than in the non-SPT group (*P* < 0.001 for all). Among the 3170 patients in the non-SPT group, 220 (6.9%) had MB in the mid-LAD, 18 (0.6%) in the distal LAD, 1 (0.03%) in the posterior descending branch of the RCA, and one each in the atrioventricular branch of the RCA and high lateral branch of the left circumflex coronary artery. Among the 23 (13.5%) out of 170 SPT patients, all MBs were located in the mid-LAD.

**Table 1:** Baseline characteristics between the patients who underwent an SPT (SPT group) and those who did not undergo an SPT (non-SPT group)

	SPT group (*n* = 170)	Non-SPT group (*n* = 3170)	*P* value
Age (year)	59.3 ± 11.5	65.3 ± 11.1	< 0.001
Male sex	109 (64.1%)	2409 (76.0%)	< 0.001
Body mass index (kg/m^2^)	23.8 ± 3.5	24.3 ± 3.8	0.078
Smoking	81 (47.6%)	1567 (49.4%)	0.650
Drinking	94 (55.3%)	1558 (49.1%)	0.118
Hypertension	84 (49.5%)	2167 (68.4%)	< 0.001
Dyslipidemia	68 (40.0%)	1875 (59.1%)	< 0.001
Diabetes mellitus	22 (12.9%)	1085 (34.2%)	< 0.001
Hyperuricemia	28 (16.5%)	1035 (32.6%)	< 0.001
Chronic kidney disease	66 (38.8%)	2096 (66.1%)	< 0.001
History of PCI	3 (1.8%)	307 (9.7%)	< 0.001
History of CABG	0	33 (1.0%)	0.181
Atrial fibrillation	9 (5.3%)	289 (9.1%)	0.089
NYHA ≥ II	4 (2.4%)	880 (27.8%)	< 0.001
Organic heart disease	23 (13.5%)	2402 (75.8%)	< 0.001
Ischemic heart disease	13 (7.6%)	1864 (58.8%)	< 0.001
Valvular disease	8 (4.7%)	543 (17.1%)	< 0.001
Cardiomyopathy	2 (1.2%)	337 (10.6%)	< 0.001
Congenital heart disease	1 (0.6%)	20 (0.6%)	0.945
Laboratory data			
Creatinine (mg/dl)	0.8 ± 0.2	1.1 ± 1.3	0.001
eGFR (ml/min/1.73 m^2^)	65.1 ± 16.5	53.1 ± 17.6	< 0.001
Uric acid (mg/dl)	5.4 ± 1.2	5.8 ± 1.5	0.001
LDL cholesterol (mg/dl)	112.1 ± 29.9	109.8 ± 32.2	0.398
HbA1c (NGSP) (%)	5.8 ± 0.7	6.2 ± 1.0	< 0.001
Medication at discharge			
Antihypertensive agents	76 (44.7%)	2170 (68.5%)	< 0.001
Beta blockers	15 (8.8%)	1081 (34.1%)	< 0.001
Calcium channel blockers	84 (49.4%)	1351 (42.6%)	0.081
Renin–angiotensin system inhibitors	28 (16.5%)	1476 (46.6%)	< 0.001
Antiplatelets	37 (21.8%)	1662 (52.4%)	< 0.001
Anticoagulants	7 (4.1%)	430 (13.6%)	< 0.001
Diuretics	1 (0.6%)	294 (9.3%)	< 0.001
Echocardiographic data			
Interventricular septum thickness (mm)	9.5 ± 1.6	10.5 ± 2.1	< 0.001
Left ventricular diameter at end- diastole (mm)	45.7 ± 4.5	47.7 ± 7.0	< 0.001
Left ventricular ejection fraction (%)	68.5 ± 6.4	62.6 ± 13.4	< 0.001
Left atrial dimension (mm)	35.1 ± 6.1	38.2 ± 7.4	< 0.001
*E*/*e*'	9.5 ± 3.1	12.4 ± 5.4	0.010
Location of MB			
Proximal LAD	0	0	–
Mid-LAD	23 (13.5%)	220 (6.9%)	0.001
Distal LAD	0	18 (0.6%)	0.325
Posterior descending branch (#4PD)	0	1 (0.03%)	0.817
Atrioventricular branch (#4AV)	0	1 (0.03%)	0.817
High lateral branch	0	1 (0.03%)	0.817

Data given as the mean ± SD or *n* (%)

*SPT* spasm provocation test, *CAG* coronary angiography, *LAD* left anterior descending coronary artery, *NYHA* New York Heart Association, *MB* myocardial bridging, *PCI* percutaneous coronary intervention, *CABG* coronary artery bypass graft, *eGFR* estimated glomerular filtration rate

### Patient characteristics between the patients with and without MB

Table 2 summarizes the baseline clinical characteristics of the present study population. The prevalence of MB was 13.9% (23 of 166 patients). All patients had chest pain or chest discomfort before the CAG in each group. Compared to the patients without MB, those with MB had higher prevalence rates of diabetes mellitus (26.1% vs. 9.8%, *P* = 0.026) and chronic kidney disease (60.9% vs. 35.7%, *P* = 0.022). There were no significant differences in terms of the age, gender, smoking, drinking, prevalence of hypertension, dyslipidemia, hyperuricemia, laboratory data, medication at discharge, and echocardiographic data except for the interventricular septum thickness (IVST) (10.2 mm vs. 9.3 mm, *P* = 0.018).

**Table 2:** Baseline characteristics of the study patients according to the presence of MB

	MB (+) group (*n* = 23)	MB (−) group (*n* = 143)	*P* value
Age (year)	58.5 ± 11.8	59.3 ± 11.5	0.779
Male sex	16 (69.6%)	89 (62.2%)	0.499
Symptoms (Chest pain or chest discomfort)	All	all	–
Body mass index (kg/m^2^)	23.9 ± 4.3	23.7 ± 3.3	0.794
Smoking	10 (43.5%)	67 (46.9%)	0.763
Drinking	10 (43.5%)	81 (56.6%)	0.239
Hypertension	9 (39.1%)	72 (50.3%)	0.318
Dyslipidemia	11 (47.8%)	54 (37.8%)	0.359
Diabetes mellitus	6 (26.1%)	14 (9.8%)	0.026
Hyperuricemia	5 (21.7%)	21 (14.7%)	0.388
Chronic kidney disease	14 (60.9%)	51 (35.7%)	0.022
Laboratory data			
Creatinine (mg/dl)	0.8 ± 0.2	0.7 ± 0.2	0.432
eGFR (ml/min/1.73 m^2^)	63.5 ± 18.9	65.3 ± 16.3	0.621
Uric acid (mg/dl)	5.7 ± 1.4	5.4 ± 1.2	0.331
LDL cholesterol (mg/dl)	112.8 ± 27.5	111.9 ± 30.1	0.900
HbA1c (NGSP) (%)	6.1 ± 0.9	5.8 ± 0.6	0.139
Medication at discharge			
Antihypertensive agents	10 (43.5%)	63 (44.1%)	0.959
Beta blockers	2 (8.7%)	12 (8.4%)	0.961
Calcium channel blockers	12 (52.2%)	69 (48.3%)	0.727
Renin–angiotensin system inhibitors	4 (17.4%)	21 (14.7%)	0.736
Antiplatelets	2 (8.7%)	33 (23.1%)	0.117
Anticoagulants	1 (4.3%)	6 (4.2%)	0.973
Echocardiographic parameters			
Interventricular septum thickness (mm)	10.2 ± 1.9	9.3 ± 1.5	0.018
Left ventricular diameter at end-diastole (mm)	45.1 ± 4.9	45.7 ± 4.4	0.625
Left ventricular ejection fraction (%)	70.9 ± 7.7	68.1 ± 6.1	0.066
Left atrial dimension (mm)	36.1 ± 5.9	34.9 ± 6.1	0.421
*E*/*e*'	8.3 ± 1.5	9.8 ± 3.2	0.378

Data given as the mean ± SD or *n* (%)

*MB* myocardial bridging, *eGFR* estimated glomerular filtration rate

### Association between the presence of MB and SPT

The SPT-positivity rate was 27.1% (45 of 166 patients). The coronary artery features derived from the SPT between the patients with and without MB are summarized in Table 3. The patients with MB had higher SPT-positivity rates than those without (56.5% vs 22.4%, *P* = 0.001). In the MB (+) group, the most frequently recorded culprit vessel for SPT-positivity was the LAD [34.8% (8/23 patients)], followed by the RCA [30.4% (7/23)] and the LCX [4.3% (1/23)]. In eight patients with SPT-positivity in the LAD, vasospasms occurred in diffuse lesions (i.e., proximal to the MB, on the MB, and distal to the MB, 1 patient), the segment proximal to the MB (5 patients), on the MB (1 patients) in the LAD, and in the diagonal branch (1 patient), respectively. SPT-positivity was more frequently recorded in patients with MB in the LAD (34.8% vs. 7.7%, *P* < 0.001), and RCA (30.4% vs. 11.2%, *P* = 0.013) than in those without (Table 3). Multivessel spasms also tended to be more often provoked in the MB (+) group [8.7% (2/23) vs. 2.1% (3/143) in the MB (−) group, *P* = 0.086].

**Table 3:** Coronary artery features of the spasm provocation test (SPT) by an intracoronary ergonovine (ER) infusion according to the presence of MB

	MB (+) group (*n* = 23)	MB (−) group (*n* = 143)	*P* value
SPT-positivity	13 (56.5%)	32 (22.4%)	0.001
Signs of ischemia			
ECG changes	13 (56.5%)	32 (22.4%)	0.001
Chest symptoms	15 (65.2%)	42 (29.4%)	0.001
Tested coronary arteries			
RCA	17 (73.9%)	127 (88.8%)	0.050
LCA	23 (100%)	143 (100%)	–
Both RCA and LCA	17 (73.9%)	127 (88.8%)	0.050
Culprit vessel of SPT-positivity			
RCA	7 (30.4%)	16 (11.2%)	0.013
LAD	8 (34.8%)	11 (7.7%)	< 0.001
LCX	1 (4.3%)	8 (5.6%)	0.806
Multivessel spasm	2 (8.7%)	3 (2.1%)	0.086

Data given as the mean ± SD or *n* (%)

*MB* myocardial bridging, *ECG* electrocardiogram, *RCA* right coronary artery, *LCA* left coronary artery, *LAD* left anterior descending coronary artery, *LCX* left circumflex artery

In the univariate logistic analysis, the presence of MB was significantly associated with SPT-positivity [odds ratio (OR) 4.509, 95% confidence interval (CI) 1.809–11.241, *P* = 0.001]. The male sex, prevalence of hypertension, chronic kidney disease, and hyperuricemia were also significantly associated with SPT-positivity (*P < *0.005, Table 4). In multivariate logistic regression analysis, the presence of MB was an independent predictor of SPT-positivity (OR 5.587, 95% CI 2.061–15.149, *P* = 0.001), when adjusted for the covariables (Table 4).

**Table 4:** Logistic regression analysis for spasm provocation test (SPT)-positivity

Variable	Univariate model			Multivariate model^a^		
	Odds ratio	95% CI	*P* value	Odds ratio	95% CI	*P* value
MB (+)	4.509	1.809–11.241	0.001	5.587	2.061–15.149	0.001
Age	1.001	0.972–1.032	0.937			
Male	3.605	1.549–8.387	0.003	3.798	1.557–9.266	0.003
Smoking	1.659	0.832–3.305	0.150			
Drinking	1.516	0.753–3.054	0.244			
Hypertension	2.113	1.048–4.262	0.037	2.637	1.214–5.729	0.014
Diabetes mellitus	1.530	0.568–4.120	0.400			
Dyslipidemia	1.191	0.594–2.390	0.622			
Chronic kidney disease	2.230	1.112–4.474	0.024			
Hyperuricemia	2.779	1.171–6.595	0.020			

*MB* myocardial bridging, *CI* confidence interval

^a^Adjusted for chronic kidney disease and hyperuricemia

## Discussion

### Major findings

The major findings of the present study were as follows: (1) in the patients with MB, the prevalence of diabetes mellitus and chronic kidney disease was more frequent, and the IVST was thicker than in those without, (2) SPT-positivity by intracoronary ER injections was more frequent in patients with MB than in those without, especially of the LAD and RCA, (3) a multivariate logistic regression analysis showed that the presence of MB was an independent predictor of SPT-positivity by intracoronary ER injections, and (4) in patients with MB, the LAD was the most frequent culprit vessel of SPT-positivity, especially proximal to the MB in the LAD. To the best of our knowledge, this is the first report to suggest a clinical relationship between MB and SPT-positivity using intracoronary ER injections.

### Frequency and positivity rate of SPT compared with the previous reports

The rate of SPT enforcement at our hospital was 170 cases in 7 years (between 2011 and 2017). According to a report examining SPT enforcement facilities in Japan [16], the number of SPT enforcements at our hospital was relatively high. In the specific population of patients who undergo intracoronary SPT, the prevalence of MB was 13.9% (23 of 166 patients) in our study. That rate was lower than that of the previous studies on SPT ranging from 31 to 36% [8, 12, 17]. The difference was evident in the SPT-positivity rate between our study and the previous studies (Table 5). The SPT-positivity rate in our study was 27.1%, which was lower than that in the previous studies of 63.4% [17], 49.0% [12], and 51.8% [8], respectively. In particular, the SPT-positivity rate in the patients without MB differed, i.e., 22.4% in our study vs. 53.4% [17], 43.3% [12], and 39.7% [8], respectively, in the previous studies. There were several possible reasons for that difference. First, our hospital specializes in cardiovascular diseases. Our SPT-positivity rate was low because SPT was performed more frequently than in the other general hospitals [16]. The second reason was the different definitions of SPT-positivity between our study and that of the previous reports. Teragawa et al. reported that SPT-positivity was defined as a ≥ 50% reduction in the arterial diameter after ACH and/or ER [8]. In contrast, the outcome of the present study was SPT-positivity as suggested by a guideline [15], namely the total or subtotal occlusion of the coronary artery with symptoms and/or significant ECG changes. Moderate-to-severe stenotic changes (50–90%) after provocation tests were not included as the SPT-positive culprit vessel in the present study. The third reason was the difference in the provocation drugs between our study and that of the previous reports. ACH and ER have been employed in SPT. While ACH was infused to evaluate the endothelial function in the previous reports [11, 12], we used ER. There has not been a definite statement as to which drugs should be infused for SPT according to our guidelines. Furthermore, because the amount of the ER injections in the present study tended to less than previously reported [18], there might have been the possibility of false negatives in the present study.

**Table 5:** SPT-positivity rate and prevalence of MB compared with the previous reports

	Definition of MB	Definition of SPT-positivity	SPT-positivity rate			Provocation drugs	Prevalence of MB (%)
			All (%)	MB (+) group (%)	MB (−) group (%)		
Arai et al	Angiographic reduction in the diameter of the coronary artery during systole, also known as the “milking effect” between systole and diastole	Total or subtotal occlusion with ECG changes and/or chest pain	27.1	56.5	22.4	ER	13.9
Teragawa et al. [8]	Angiographic reduction in the diameter of the coronary artery during systole of > 15%	≥ 50% reduction in the arterial diameter, associated with ECG changes and/or chest pain	51.8	73.2	39.7	ACH and ER	36.0
Saito et al. [12]	Angiographic systolic narrowing of coronary vessels that was pronounced than in neighboring normal vessels, with partial or complete decompression during diastole (i.e. milking effect)	Total or subtotal occlusion with ECG changes and/or chest pain	49.0	59.3	43.3	ACH	35.7
Xiang et al. [19]	50% angiographic reduction in the diameter of the coronary artery during systole	90% or above reduction in the arterial diameter with ECG changes and/or chest pain	63.4	85.3	53.4	ACH	31.5

*SPT* spasm provocation test, *MB* myocardial bridging, *ECG* electrocardiogram, *ER* ergonovine, *ACH* acetylcholine

### Frequency and characteristics of the patients with MB

The prevalence of MB in the CAG in the present study was 7.3% (243 of 3340 patients), which kept with the results of the other studies that have shown an MB prevalence ranging from 0.5 to 12% [1, 2] in the CAG. Similar to the previous reports in Japanese patients [8, 12], 166 patients in the SPT group were relatively young (55–63 years) but there was no significant age-difference with regard to the prevalence of MB (58.5 vs. 59.3, *P* = 0.779). We found an interesting observation that the prevalence of diabetes mellitus and chronic kidney disease was higher, and the IVST thicker in the patients with MB than in those without. Diabetes mellitus and chronic kidney disease have been known to be associated with endothelial dysfunction [19, 20], and thus, those comorbidities might have interacted with the MB for coronary spasms. Nonetheless, the predictive values of those comorbidities for the SPT-positivity were not independent. Although it is speculative, a thicker IVST in the patients with MB might be related to endothelial dysfunction due to a stronger coronary compression in the MB segment, leading to an increase in the mechanical stimulation effect at the MB segment, as discussed below.

### Possible mechanisms between MB and coronary spasms

In the present study, the SPT-positivity by intracoronary ER injections was more frequent in patients with MB than in those without. Even after a multivariate adjustment, the presence of MB remained to be an independent predictor of SPT-positivity. The reported causes of coronary spasms are abnormal responses of the autonomic nervous system [21], endothelial dysfunction [22], abnormal or hyper-reaction of vascular smooth muscles [23], inheritance [24], and the specific anatomy of the coronary arteries [9, 12]. In patients with MB, the reasons why MB is associated with coronary spasms are as follows: (1) the mechanical stimulation effect of MB causes longstanding mechanical stress in MB segments, leading to focal endothelial dysfunction [8] and (2) a structural lumen obstruction in MB segments causes hemodynamic and wall shear stress (WSS) changes [25], leading to endothelial dysfunction [26, 27]. Coronary compression by MB results in a relatively low WSS proximal and distal to the MB segment and a high WSS in the MB segment. Similar to the previous reports [7–9, 12], the culprit vessel that was SPT-positive was most frequently located in the LAD, especially proximal to the MB segment in the LAD [12], suggesting endothelial dysfunction of that artery.

In general, the mechanism of coronary spasms may not be derived from only one cause, rather there may be several factors. Ohyama et al. reported that perivascular components, including perivascular adipose tissue and adventitial vasa vasorum caused an increased activity of Rho-kinase, leading to coronary spasms [28, 29]. The epicardial adipose tissue surrounding the LAD has been reported to be more directly linked to cardiovascular events than EATs surrounding the LCX and RCA [30]. Masuda et al. reported that endothelial nitric oxide synthase and endothelin-1, reflecting the endothelial function, were significantly lower in MB segments [31]. The difference in the expression of these vasoactive agents in patients with MB may influence the microvascular function remotely, leading to not only focal (epicardial), but also microvascular endothelial dysfunction [11]. These mechanisms may cause that the culprit vessel of the SPT-positivity was recorded not only in MB segments in our study, even though the MB segments also tended to be spastic and suggested a certain degree of endothelial dysfunction in these patients (Fig. 3). Saito et al. reported that patients with MB had a higher SPT-positivity not only in the LAD (61% vs. 39%, *P* < 0.001) but also in the RCA (37% vs. 25%, *P* = 0.020) by intracoronary ACH testing [12], even using intracoronary ER testing, as we found in our study.This was also reinforced by our finding that multivessel spasms also tended to be more frequently provoked in the MB (+) group than MB (−) group (*P* = 0.086). The incidence of multivessel spasms in the MB (+) group might have been further increased because ER provocation testing could not be performed in the RCA more frequently in this group [26.1% (6/23) vs. 11.2% (16/143), *P* = 0.050] when the LCA became SPT-positive with persistent ischemic ECG changes and/or chest pain that required nitrate injections to relieve the vasospasms.

### Limitations

The present study had several limitations. First, this was a single-centre retrospective observational study, the sample size was rather small, and patient selection bias was present, which might have limited the generalizability of the present study. Nonetheless, a younger age and less comorbidities and cardiovascular risks in the patients who underwent SPT are generally well-known in routine clinical practice. Second, MB was defined by only the angiographic characteristics [32], and we did not perform other modalities, including coronary computed tomography angiography, positron emission tomography, or contrast stress echocardiography [33, 34]. Third, our analyses could not evaluate the length or depth of the MB segment, even though the hemodynamic impact of the MB depends on the thickness and length of the bridge [34]. Fourth, we could not perform SPT of the RCA in 6/13 cases among the patients with MB and in 16/32 cases among those without MB. Therefore, the number of SPT-positivity cases in the RCA might have been underestimated. Finally, we used only ER injections for the SPT, and the dose of the ER was lower than that used in the previous reports [18].

## Conclusion

The presence of MB was an independent predictor of SPT-positivity in patients with chest pain without a significant obstruction of the coronary arteries. The patients who had MB on CAG might be carefully observed because coronary spasms could be the cause of their symptoms.
